# Supramolecular Self-Healing Sensor Fiber Composites for Damage Detection in Piezoresistive Electronic Skin for Soft Robots

**DOI:** 10.3390/polym13172983

**Published:** 2021-09-02

**Authors:** Antonia Georgopoulou, Anton W. Bosman, Joost Brancart, Bram Vanderborght, Frank Clemens

**Affiliations:** 1 Department of Functional Materials, Empa—Swiss Federal Laboratories for Materials Science and Technology, 8600 Dübendorf, Switzerland; 2 Brubotics, Vrije Universiteit Brussel (VUB) and imec, B-1050 Brussels, Belgium; Bram.Vanderborght@vub.be; 3 SupraPolix BV, 5612AX Eindhoven, The Netherlands; bosman@suprapolix.com; 4 Physical Chemistry and Polymer Science (FYSC), Vrije Universiteit Brussel (VUB), B-1050 Brussels, Belgium; joost.brancart@vub.be

**Keywords:** self-healing thermoplastic elastomers, self-healing sensors, strain sensor, electronic skin, soft robotics

## Abstract

Self-healing materials can prolong the lifetime of structures and products by enabling the repairing of damage. However, detecting the damage and the progress of the healing process remains an important issue. In this study, self-healing, piezoresistive strain sensor fibers (ShSFs) are used for detecting strain deformation and damage in a self-healing elastomeric matrix. The ShSFs were embedded in the self-healing matrix for the development of self-healing sensor fiber composites (ShSFC) with elongation at break values of up to 100%. A quadruple hydrogen-bonded supramolecular elastomer was used as a matrix material. The ShSFCs exhibited a reproducible and monotonic response. The ShSFCs were investigated for use as sensorized electronic skin on 3D-printed soft robotic modules, such as bending actuators. Depending on the bending actuator module, the electronic skin was loaded under either compression (pneumatic-based module) or tension (tendon-based module). In both configurations, the ShSFs could be successfully used as deformation sensors, and in addition, detect the presence of damage based on the sensor signal drift. The sensor under tension showed better recovery of the signal after healing, and smaller signal relaxation. Even with the complete severing of the fiber, the piezoresistive properties returned after the healing, but in that case, thermal heat treatment was required. With their resilient response and self-healing properties, the supramolecular fiber composites can be used for the next generation of soft robotic modules.

## 1. Introduction

Soft electronics, such as sensors, find applications in many rapidly growing fields, such as soft robotics, wearable devices and electronic skin [1,2,3,4,5,6,7,8]. However, soft electronic devices are prone to damage, especially when they experience large mechanical deformation or harsh environments [9]. Introducing a self-healing ability into soft electronic materials and devices can increase the durability and reliability of the system, and therefore prolong the self-life of the device [10,11].

Autonomous intrinsic self-healing materials do not require an external healing agent or trigger, and their mechanism is based on reversible physical or chemical intramolecular interactions. For autonomous intrinsic self-healing elastomers, the healing mechanism and the restoration of the properties are based on dynamic covalent [12,13,14] or non-covalent bonds [15]. Non-covalent self-healing elastomers possess reversible bonds, and as a consequence, they can adapt to environmental changes, and are recyclable and compatible with facile processing methods [16]. A popular option for self-healing elastomers using non-covalent bonds are the supramolecular networks based on hydrogen bonding [17,18,19]. The advantage of hydrogen bonding interactions is that they can be reformed very quickly, often leading to the autonomous healing of damage, without the need for an external trigger to activate the healing action. A review article on self-healing materials was published by Terryn et al. in 2021, and shows the relevance of the self-healing materials for soft robotic applications [20].

Hydrogen-bonded self-healing elastomers can be combined with conductive fillers for the development of conductive composites [21,22]. The use of carbon fillers for the development of self-healing strain sensors has been proven in the past to be a useful strategy [23,24,25,26,27,28]. A high concentration of the carbon filler will improve the conductivity of the composite; unfortunately, a high filler content will cause stiffening and impair the self-healing ability of the polymer matrix material [24]. Yan et al. investigated a polysiloxane/carbon black-based self-healing elastomer, and they achieved good conductivity, but only up to 30% strain [29]. In self-healing conductive systems, not only the mechanical, but also the electrical properties should be restored after the healing process [21,25]. After healing, the electrical properties may be altered since the molecular structure of the polymer might be different, forcing the conductive network to rearrange [23]. Cao et al. developed a flexible strain sensor, based on a self-healing hydrogen-bond based polymer with carbon nanotubes, with a rapid healing behavior; unfortunately, the effect of dynamic cycling movement on resilient sensor behavior was not investigated [30]. A summary of carbon-based elastomer sensor materials with integrated self-healing properties has been made by Georgopoulou and Clemens [31]. As an alternative to carbon-based fillers, Tee et al. developed a self-healing conductive composite based on metallic particles, and a supramolecular elastomer for tactile and flexion sensor electronic skin applications [32]. Even though their system exhibited a rapid self-healing response and good piezoresistivity, they did not investigate the dynamic behavior. For conductive self-healing elastomers, it remains a big challenge to combine good mechanical properties, such as strength and elasticity, with good self-healing properties and multi-sensing abilities, such as sensing deformation and damage at the same time [17,33,34].

Besides monitoring the stress–strain behavior profile of the undamaged material, self-healing piezoresistive sensors can also be used for monitoring the structural integrity of materials [35,36]. For complex soft material structures, self-healing conductive materials can be used for the detection of damage, even if it is not visible with the naked eye [23]. Additionally, it has been demonstrated that strain sensors can identify healing processes and localize the damaged area [37,38]. Pu et al. developed a piezoresistive sensor based on carbon black and self-healing polyurethane with Diels-Alder bonds to detect the formation of cracks via the readout of the resistance change. However, their composite exhibited only low strain up to the point of fracture (30%), compared to other elastomer-based piezoresistive sensors [39]. In particular, a high strain at the point of fracture is critical for successful future integrations into soft robotic modules.

In this work, a novel self-healing composite with piezoresistive elastomer-based strain sensor fibers was developed, by combining a quadruple hydrogen-bonded (UPy) supramolecular elastomer [18,40] with carbon black filler. To avoid the stiffening of the resulting self-healing skin structure, the sensor fiber composite approach reported by Georgopoulou et al. was used [41,42]. Self-healing sensor fiber composites were developed to monitor strain and damage inside the material. The composites were able to endure elongations of up to 100%, which is higher than already reported in the literature for self-healing damage detection sensors. Great elongation at the point of fracture is essential for soft actuator modules in robotics. To be able to identify the potential of such materials for soft robotic modules in the future, a fundamental study on self-healing sensor fibers (ShSF), self-healing sensor fiber composites (ShSFC), and the integration of two soft robotic bending actuator modules, is featured in this work. The successful recovery of the sensing performance of the ShSFC after damage and healing was investigated.

## 2. Materials and Methods

### 2.1. Self-Healing Compounds

#### 2.1.1. Carbon Black-Loaded UPy-1 and Sensor Fiber Preparation

**Figure 1:**
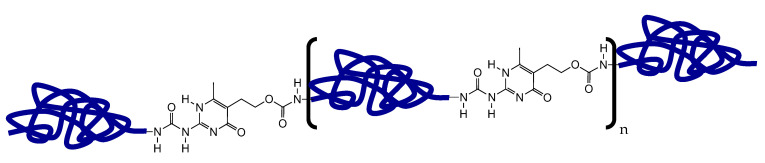
Schematic representation of the UPy-elastomers; in polymer UPy1, the blue line depicts the hydrogenated polybutadiene backbone extended with 4′-methylenebis(cyclohexyl isocyanate), and in polymer UPy2, the blue line depicts the poly(tetramethylene oxide) backbone extended with isophorone diisocyanate.

According to Mattmann et al., a high content of carbon filler is required to achieve elastomer fibers with sufficient piezoresistive sensor properties [43]. Therefore, the UPy-elastomer 1 was designed to develop an elastomer composite with a high carbon filler content. The self-healing supramolecular UPy-elastomer 1 was obtained as described previously from 2-amino-4-hydroxy-5-hydroxyethyl-6-methyl-pyrimidine, 4,4′-methylenebis (cyclohexyl isocyanate) and hydrogenated polybutadiene end-capped with hydroxyl groups (M_n_ = 2000) [40]. A schematic representation of the UPy-elastomer can be seen in Figure 1.

The resulting self-healing supramolecular elastomer was subsequently dissolved in a chloroform/ethanol mixture with 10% *v/v* ethanol concentration, and conductive carbon black powder was added (Ensaco 260G, Timcal, Bodio, Switzerland) in a weight ratio of 1:2.32 (carbon black: Polymer). In the next step, the solvent was removed by evaporation, resulting in heterogeneous rubbery solids comprising 30 wt.% carbon black. These solids were cut into pieces and fed into a Haake Minilab twin-screw extruder (Thermo Fisher Scientific, Karlsruhe, Germany) operating at 140 °C, resulting in a self-healing conductive composite that could be used for sensor fiber preparation via the extrusion method.

For ShSF fabrication a capillary rheometer Rh7 (Netzsch Gerätebau GmbH, Selb, Germany) was used. This process for the fabrication of piezoresistive fiber sensors based on thermoplastic elastomers has been described elsewhere [7,44]. The extrusion was performed at a temperature of 95 °C with a 0.5 mm die. Finally, a supramolecular ShSF with a diameter of 0.5 mm was obtained after the extrusion process. After the production, the supramolecular fibers were stored for three days before further processing and testing.

The concentration of the CB for the ShSF was selected at 30 wt.% because sensor fibers with a filler content of 25 wt.% were not conductive, and composites with a higher filler content than 30 wt.% could not be extruded with a constant diameter because of the melt fracture effect. Melt fracture occurs when excessive shear stress is exerted on a melt, leading to roughness in the extrudate [45,46]. This phenomenon is also known as sharkskin, and it was observed at a low shear rate.

#### 2.1.2. UPy-2 and Matrix Film Preparation

The UPy-elastomer 2 was designed to recover 100% mechanical properties, after the self-healing process at room temperature. The self-healing supramolecular elastomer UPy-2 was obtained as described previously from 2-amino-4-hydroxy-5-hydroxyethyl-6-methyl-pyrimidine, isophorone diisocyanate and poly(tetramethylene oxide) (M_n_ = 1000) [40]. The isolated polymer was subsequently processed into a clear film with a thickness of 0.7 mm using a hydraulic laboratory hot press (Fontijne Press, Delft, The Netherlands) at 120 °C and 150 N.

### 2.2. Fabrication of Fiber Composites Using the Lamination Method

**Figure 2:**
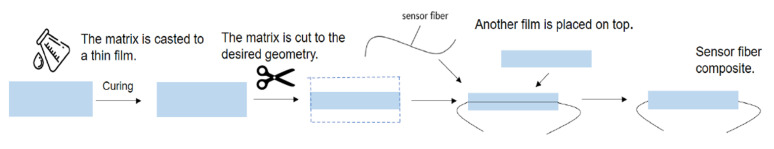
Steps for making ShSFC using the lamination method.

A lamination process was used to embed the UPy-1 self-healing piezoresistive ShSF into the self-healing UPy-2 matrix (Figure 2). The elastomeric film, as obtained after hot-pressing, was cut into the desired rectangular shape with dimensions of 130 × 10 mm. Subsequently, the supramolecular ShSF was placed between two cut films. Therefore, the composite had a thickness of 1.4 mm. The supramolecular elastomer layers were left in contact for one hour. During the contact, the two films fused together by exchanging hydrogen bonds across the contact surfaces, embedding the SHSF in the middle of the laminated structure.

### 2.3. Characterization of Self-Healing UPy-Elastomers with and without Carbon Black Filler

The molecular weight was measured in THF against polystyrene standards using a Shimadzu LC-10DVP system (Shimadzu, Kyoto, Japan), equipped with a Shimadzu RID-10A detector and a PLgel 5 um mixed-D column.

### 2.4. Tensile Testing and Characterization of the Mechano-Electrical Behavior

The tensile testing of UPy-1 and UPy-2 was performed using a Shimadzu AGS-X (Kyoto, Japan) tensile tenser equipped with a 500 N load cell and operated at a crosshead speed of 50 mm/min, resulting in a strain rate of 2.27 min^−1^. The samples had a dog bone shape geometry with thicknesses of 1 mm. The Young’s modulus was calculated from the slope of the stress–strain plot, between 0 and 1% strain.

For the investigation of the piezoresistive response of the ShSF and the ShSFC, tensile tests were performed with the simultaneous recording of the electrical resistance. For the tensile testing, a Z005 tensile testing machine (ZwickRoell, Ulm, Germany) with a 200 N load cell was used. In order to minimize the slipping of the test samples, pneumatic clamps with a pressure of four bars were used. The gauge length of the sample was 50 mm. Tensile tests were performed at a constant deformation rate of 200 mm/min, resulting in a strain rate 4 min^−1^. Simultaneously, the electrical resistance was investigated using a Keithley 2450 multimeter (Keithley Instruments, Solon, Ohio, USA). The current of the signal was measured, while a constant voltage of 1 V was applied to the ShSF. A sampling rate of 10 Hz was selected, and the relative resistance (*R_r__el_*) was calculated using the following formula:

(1) $$R_{rel}=\frac{R-R_{0}}{R_{0}}$$

where *R* is the value of the resistance and *R*_0_ the initial value of the resistance at the beginning of the test (no strain applied to the sensor).

### 2.5. Fabrication of Soft Robot Modules for Application Tests

**Figure 3:**
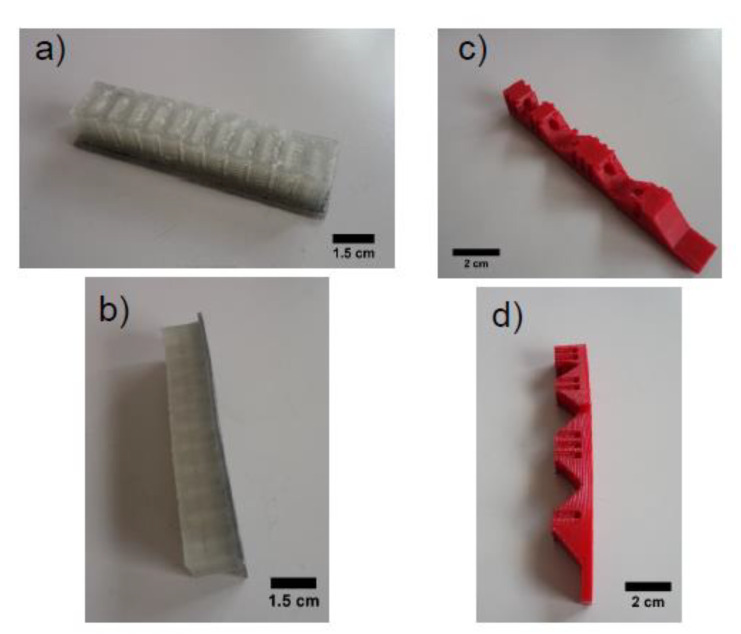
The two bending actuators produced with fused deposition modeling (FDM): a pneumatic bending actuator (**a**) top view and (**b**) side view and a tendon-driven actuator (**c**) top view and (**d**) side view.

In this work, two different soft robotic modules were used for testing the applicability of the ShSFC as a piezoresistive electronic skin. The two soft robotic bending modules (Figure 3) exerted compression and tension on the skin, respectively.

The two bending actuators (Figure 3a,b) were produced by fused deposition modeling, which is an extrusion-based method of additive manufacturing. For the pneumatic bending actuator, a mold design for silicone rubber casting was modified [47]. The pneumatic bending actuator was printed with a pellet-based FDM 3D printer, equipped with a single screw extruder, as described in an earlier study [48]. The function of the actuator is based on a network of pneumatic channels (PneuNets). A SEBS thermoplastic elastomer, compatible with FDM, was used with shore hardness 18A from Kraiburg (Waldkraiburg, Germany). The maximum deformation exerted on the ShSFC embedded in the robotic modules was calculated at 50%.

As for the tendon-based actuator (Figure 3c,d), the fabrication was performed with filament-based fused deposition modeling. A TPU (thermoplastic polyurethane)-based thermoplastic elastomer with the commercial name NinjaFlex (Ninjatek, Manheim, PA, USA) was used. The fabrication of the tendon-based actuator was performed at 220 °C. The actuation was tendon-based, and a servomotor was used for moving the tendon. A maximum deformation of the tendon-based actuator of 50%, based on the change in length of the tendon, was calculated.

### 2.6. Fabrication and Attachment of the Sensorized Electronic Skin, Based on the Fiber Composites on the Soft Robots

**Figure 4:**
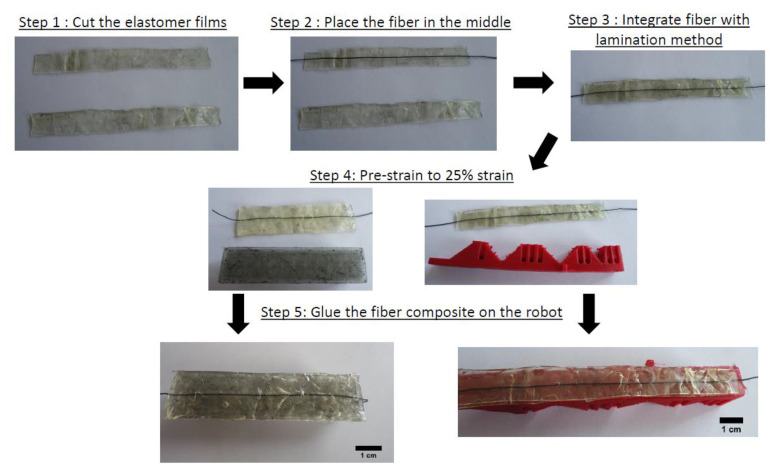
Schematic description of the ShSFC fabrication steps for the electronic skin and the attachment on the soft robot.

Based on the tensile testing results of the single ShSF and the results, reported by Georgopoulou et al. [41], a pre-straining of 25% for the ShSFC was performed before attaching on the soft robotic structures. For the fixation of the ShSFCs, an adhesive glue Sil-Poxy (Smooth-On, Mangungie, PA, US) was used. Sufficient adhesive is required to ensure that the pre-straining conditions are maintained, even after the fiber is cut. Figure 4 shows the different steps of the fabrication of the sensorized skin and the fixation on the soft robotic modules.

### 2.7. Self-Healing Protocol and Assessment

In order to test the self-healing capabilities of the material, two different cases were investigated. In one case, only the elastomeric matrix was cut, but the sensor fiber remained intact. The healing was performed at room temperature. In the other case, the matrix and the fiber were cut and the healing was performed at 90 °C for 10 min. In both cases, the electrical signal was recorded after the damage and after the healing using a Keithley 2450 multimeter. The properties of the damaged and healed systems were assessed following the procedures detailed in Section 2.4 for the characterization of the pristine fiber composites.

## 3. Results

### 3.1. Tensile Testing for the UPy-1 and UPy- 2

The UPy-1 is composed of an apolar polymer chain, extended by UPy-moieties with an average molar mass (M_n_) of 40 kDa and a mass average molar mass (M_w_) of 79 kDa. For the UPy elastomer 2, the polymer chain consisted of UPy-moieties with a M_n_ of 18 kDa and a Mw of 31 kDa. Comparing the mechanical properties between elastomers UPy-1 and UPy-2, it can be seen that the UPy-2 elastomer had a higher Young’s modulus and tensile strength before damage (Table 1). Using carbon black as a filler for the UPy-1 led to a significant increase in Young’s modulus and strength. However, the strain at the point of fracture decreased from 1200% to 70%.

In addition to the initial mechanical properties, the self-healing capabilities of the two elastomers and the UPy-1 composite after four hours at room temperature are shown in Table 1. It can be seen that in the case of elastomer UPy-1, only 75% of the tensile strength and 60% of the strain at the point of fracture could be achieved after the self-healing process. As expected, a high filler content of 30 wt.% CB resulted in a limited recovery of the mechanical properties after the self-healing step at room temperature: 73% of the tensile strength and 37% of the strain at fracture were recovered. Due to the high content of carbon filler, the mobility of the polymer chains decreased and the probability of the reformation of broken network chains was hindered. The mechanical properties of elastomer UPy-2 underwent a full recovery after 4 h of healing at room temperature.

### 3.2. Characterization of the Self-Healing Sensor Fiber (ShSF)

**Figure 5:**
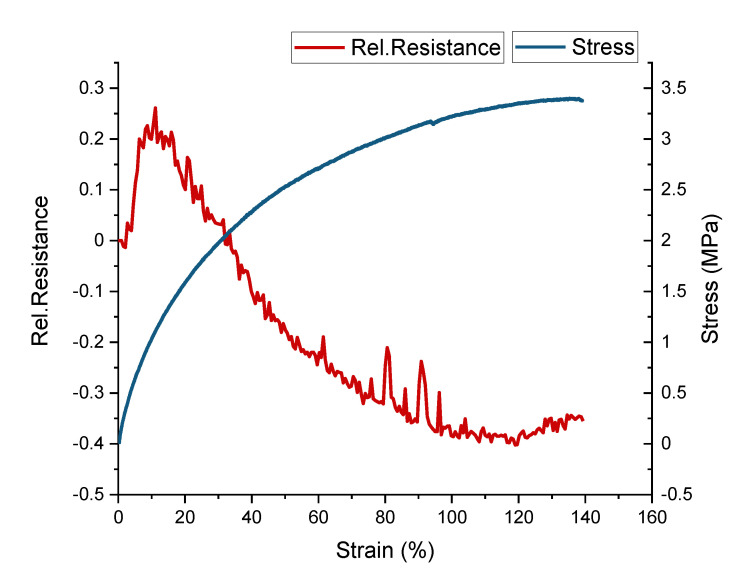
Mechanical and electrical behavior of the ShSF during a tensile test to the point of fracture.

The self-healing sensor fiber (ShSF) was created using the conductive composite composed of the UPy elastomer 1 and 30 wt.% carbon black filler. From Figure 5, it can be seen that the ShSF exhibited different piezoresistive responses in different strain areas.

Interestingly, the strain at fracture here was two times higher than in the case of the UPy-1 with 30 wt.% carbon black filler, shown in Table 1. It is suggested that in the case of the ShSF, the change in the alignment of the carbon filler is caused by the additional processing step of extruding the composite into a fiber with a small diameter (0.5 mm). The Young’s modulus for the ShSF was 17 MPa, and the ultimate tensile strength was 3.4 MPa.

Looking at the sensor behavior in Figure 5, it can be observed that the relative resistance initially increased (positive piezoresistive sensor response) until 10% strain, at which point it reached its maximum. Up to 120% strain, a negative piezoresistive response could be observed. Further straining, until the fracture of the fiber, resulted in a second increase in the relative resistance (positive piezoresistive sensor effect). This behavior is well known for piezoresistive strain sensors based on thermoplastic elastomers, and has been described in detail by Flandin et al. [49] and others [41,43,50]. Culha et al. mentioned that a reduction in piezoresistive sensor signal can be avoided by increasing the content of the conductive filler [50]. The first part of the curve, where the relative resistance was increased, is called the initiation phase, and this is caused by a rearrangement of the conductive network, formed by the conductive filler. In Figure 5, the initiation phase extends up to 10% strain. The second phase is the so-called reversible phase, where the relative resistance decreases with the increasing strain, and in Figure 5, this phase extends up to 120%. The last phase is known as the recoverable damage phase, and during that phase, the slope of the curve of relative resistance/strain becomes positive. In Figure 5, the recoverable damage phase lasts until the point of fracture (140% strain). It has been demonstrated in the past that by applying strain in the range of the reversible phase, a sensor response with low drift and relaxation behavior can be achieved, even though the sensor will exhibit a negative piezoresistive effect [51]. Over the full measurement range, the electrical signal of the ShSF was hampered by significant noise, as can be seen in Figure 5. This noise could be attributed to interactions between the conductive fiber and its environment, as discussed by Jynno et al. and Kitora et al., for this effect has sometimes been observed in conductors [52,53].

**Figure 6:**
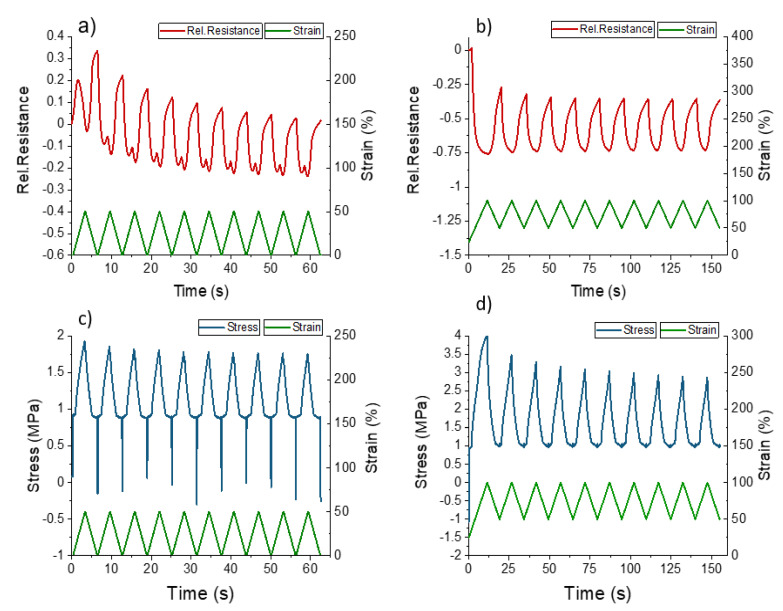
Response of the mechanical stress and electrical signal during dynamic tensile testing of the ShSF between 0 and 50% (**a**,**c**) and 50 and 100% strain (**b**,**d**).

In addition to the single tensile test, the response of the ShSF during dynamic testing can be seen in Figure 6. The applied strain varied between 0 and 50%, based on the deformation calculated for the robotic tendon-based actuator used in the study. The resistance of the ShSF signal was decreased by increasing the strain (Figure 6a). As expected following the results in Figure 5, the ShSF exhibited reverse piezoresistivity during the dynamic testing.

Looking at the first cycles in Figure 6a, it can be seen that the relative resistance first increased and then decreased, in line with the tensile test to the point of fracture in Figure 5. Later on, the sensor data become difficult to interpret because of the buckling effect at 6% elongation, as shown in the stress curve in Figure 6c. This buckling behavior is caused by the viscoelastic behavior of the material. This effect is well known for elastomeric materials, and depending on the stiffness, causes zero or negative values of stress. A change in the slope of the electrical signal from negative to positive was observed in the loading part after the second cycle at 46% (Table 2). A change in the negative part of the slope was also seen with around 6% strain. The significant drift in the electrical sensor signal (110% between the 2nd and 10th cycles) was assessed via the dynamic test. Based on these results, a pre-straining strategy, as described by Georgopoulou et al. [41,42], in order to improve the sensor behavior, was used. Indeed, pre-straining the ShSF to 50% strain significantly improved the sensor response, as shown in Figure 6b,d. By pre-straining, the drift during the different cycles could be significantly reduced from 110% to 2% (Table 2).

### 3.3. Characterization of the Self-Healing Sensor Fiber Composites (ShSFC)

**Figure 7:**
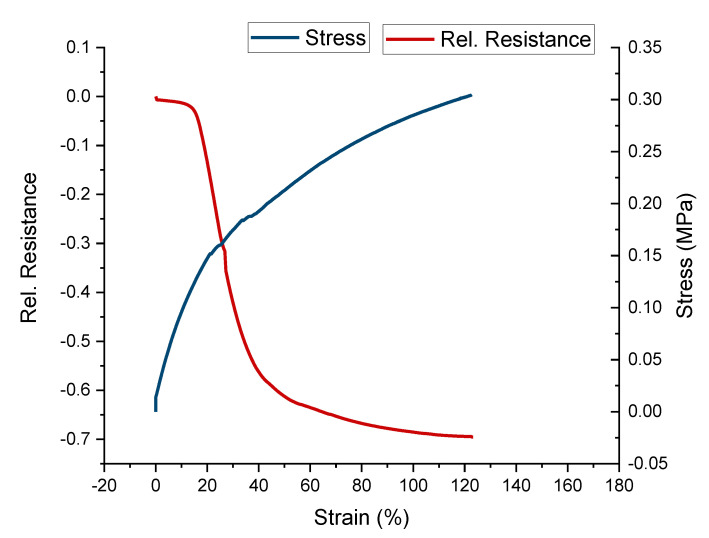
Mechanical behavior and the response of the electrical resistance during a tensile test to the point of fracture for the ShSFC.

For developing self-healing sensor fiber composites (ShSFC), a self-healing supramolecular elastomer was used as a matrix material (UPy elastomer 2). This step was necessary, since the ShSFC would later be used as the sensorized electronic skin to be used on soft robotic modules. Since the two self-healing elastomers were both composed of the same self-complementary hydrogen-bonding units, a strong interface between sensor fiber and matrix was expected [54]. Because of the small diameter of the ShSF, aligning the two parts when the fiber is completely severed will be challenging. Therefore, integration in the matrix will help to achieve the alignment of the two sides. Embedding the fibers in a matrix can alter the sensor response, and for that reason, mechano-electrical characterization has been performed for the ShSFC. The corresponding electrical and mechanical responses to strain are displayed in Figure 7, wherein the sample was strained until fracture.

The electrical resistance response shows that the ShSFC exhibited a negative piezoresistive effect until the point of fracture. It is worth mentioning that, at the strain whereat the ShSF changed from exhibiting a negative to a positive piezoresistive behavior, the composite broke. At low strains, the relative resistance decreases slightly up to 17%, followed by a steep decrease in the relative resistance until about 44%. This range is quite similar to what has been observed for a single fiber. Remarkably, the signal-to-noise ratio of the electrical signal was strongly improved in the embedded fiber, when compared to the tensile testing to the point of fracture of the single fiber. We assume that the elastomeric matrix helps to protect the sensor fiber from environmental effects.

**Figure 8:**
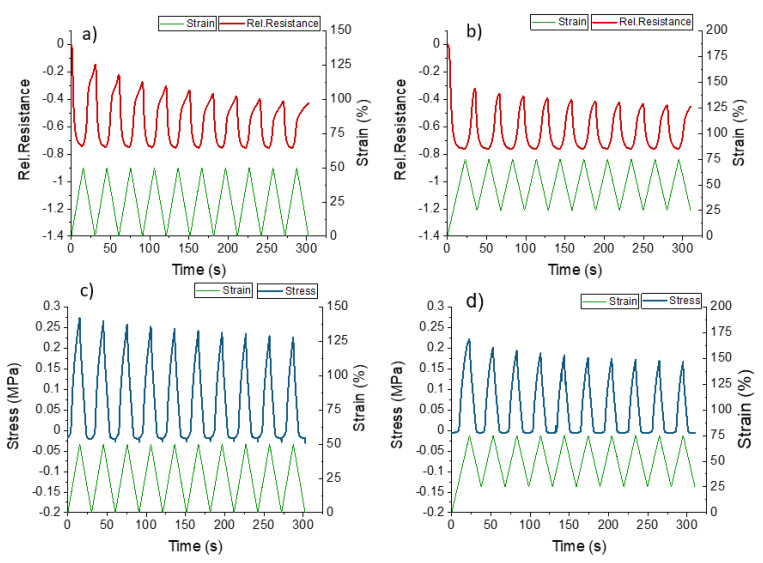
Response of the electrical and mechanical behavior during dynamic tensile testing for ShSFC between the strains 0 and 50% (**a**,**c**) and 25 and 75% (**b**,**d**).

For the evaluation of the dynamic electrical and mechanical response, a cyclical tensile test was performed on the ShSFC (Figure 8).

As shown in Figure 8, the ShSFC responds with a monotonic increase and decrease in the electrical signal during the loading and unloading phases, respectively. Therefore, embedding the ShSF in the matrix effectively removed this undesired electrical signal change. Without pre-straining, a drift of 63% and bucking at 24% strain can be observed. Due to the reduced point of fracture, it was decided that pre-straining of 25% will be employed for the dynamic testing. Comparing the results for the ShSFC with and without pre-straining (Table 3), it can be seen that the drift of the electrical signal could be decreased from 63% to 22%, which is less than the reduction observed for the ShSF. In contrast to the single ShSF, the buckling here appeared at higher strain values. Based on these results, it can be assumed that the matrix material shows much higher viscous behavior compared with the pure ShSF.

Overall, the electrical noise could be eliminated and the sensor behavior could be improved by embedding the ShSF into a self-healing elastomeric matrix. Pre-straining the ShSFC helped to reduce the drift of the electrical signal between different cycles, but the drift was still large. Further investigations were therefore carried out on pre-strained ShSFC piezoresistive electronic skins for the in situ monitoring of soft robotic actuator modules.

### 3.4. Application: ShSFC-Based Sensorized Skin for Soft Robotic

Electronic skin with self-healing properties can contribute to prolonging the life of soft robot skin structures, by way of structural health monitoring and the repair of the mechanical properties and sensory features. The strain-sensing properties of the piezoresistive ShSFC, used as an electronic skin, were evaluated before damage, after damage, and after the self-healing process. In all the experiments with the robotic modules, the electronic skin was pre-strained before being attached to the robotic module. Instead of tensile testing, the bending behavior of the electronic skin was investigated on two different soft robotic actuator modules. In the case of the tendon-based actuator, the ShSFC was operated under tension mode, and the pneumatic-based actuator was operated under compression mode.

**Figure 9:**
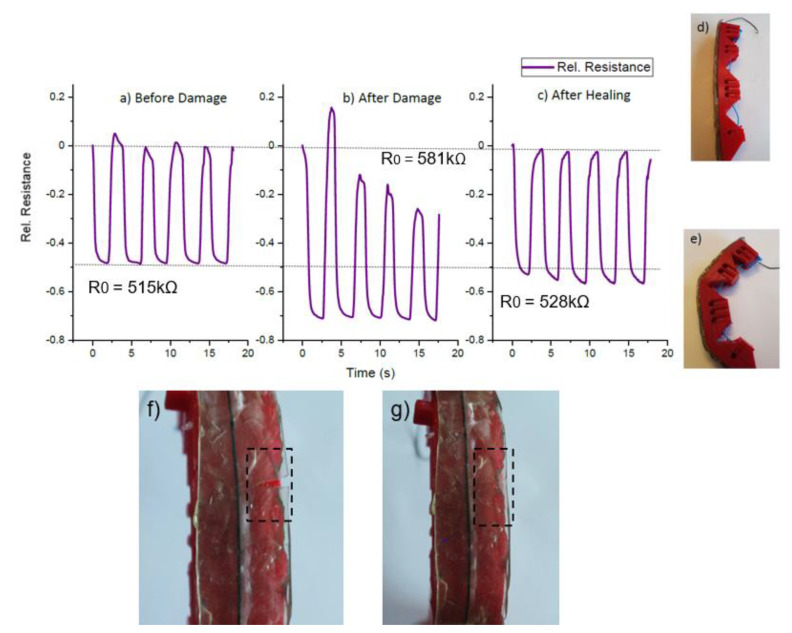
The electrical signal from the piezoresistive sensor in the skin during the dynamic bending (0–50% tension based on the change in the length of the tendon) for the tendon-based soft actuator in five repeated cycles (**a**) before and (**b**) after partial damage of the ShSFC matrix, and (**c**) after the healing of the ShSFC matrix. (**d**) The tendon-based soft actuator in the initial position and (**e**) in a bent position. The tendon based-soft actuator with (**f**) partially damaged and (**g**) healed ShSFC-based skin.

To investigate the damage detection capacity of the ShSFC-based electronic skin, dynamic bending tests were performed on the partially damaged matrix (Figure 9f). In Figure 9, due to the servomotor control, a short static interval (2 s) can be seen in the measurement. During this short static part, a small relaxation of the electrical signal can be observed.

Looking at the sensor signal response before the damage (Figure 9a), it can be seen that during cycling, the relative resistance changed from 0 to −0.5, with a small drift at the end of each cycle. In order to assess the damage on the electronic skin, the matrix was partially cut with a knife (Figure 9f) while keeping the piezoresistive fiber intact. Figure 9b shows the sensor signal after partially cutting the matrix. The relative resistance showed a significant drift after each cycle and a higher change in relative resistance (−0.7). Because of the lower geometrical stiffness in the partially cut part, a higher relative resistance value can be observed. Due to the lower geometrical stiffness, greater elongation of the sensor fiber in the damaged area can be expected. The greater elongation of the ShSF will result in a higher relative resistance change, as shown in Figure 9b. Based on this result, we can assume that the ShSFC electronic skin is suitable for detecting damage of the matrix. We suggest that for real-life applications, a threshold, for example at −0.7 relative resistance, can be programmed to detect possible damage to the elastomeric matrix of the ShSFC. Subsequently, the cut elastomeric matrix was allowed to self-heal at room temperature for 10 min (Figure 9g), and the resulting signal behavior is shown in Figure 9c. Remarkably, the initial sensor response was fully regained after the healing process. The small difference in the relative resistance values between 9a and 9c can be explained by the viscoelastic effect of the UPy-2 matrix, as already discussed previously, and a re-calibration of the sensor after healing is necessary. As for the actual resistance, expressed by *R*_0_, the value increased from 515 kΩ to 581 kΩ. This increase could be again explained by the change in the geometrical stiffness in the damaged area. After healing, the resistance returned to the range of the original value (523 kΩ).

**Figure 10:**
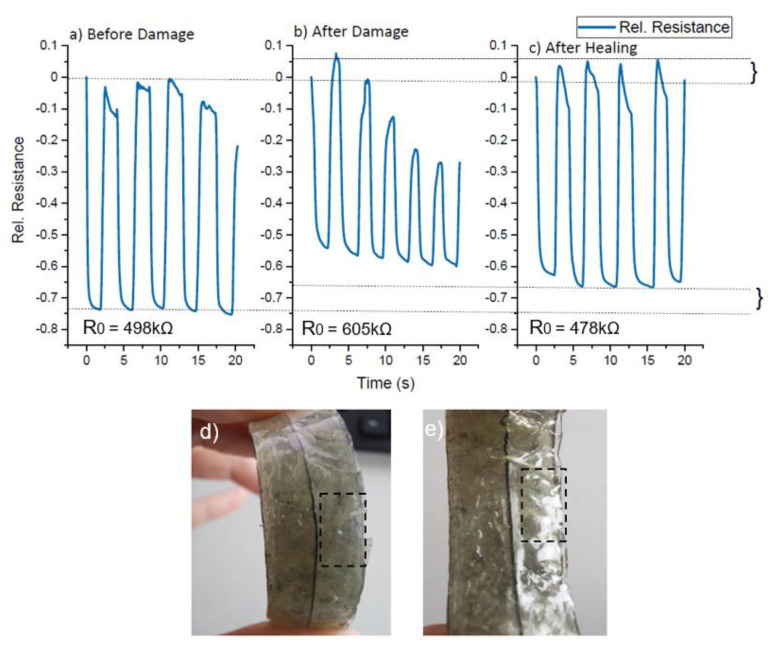
The electrical signal from the piezoresistive sensor in the skin during the bending motion (change between 0 and 90° bending angle) of the tendon-based soft robotic actuator in five repeated cycles (**a**) before and (**b**) after partial damage of the ShSFC matrix and (**c**) after the healing of the ShSFC matrix. The pneumatic-based bending actuator with (**d**) partially damaged and (**e**) healed ShSFC matrix.

For the soft pneumatic bending actuator module, the ShSFC electronic skin was operated under compression mode (Figure 10). Similarly to the tendon-based actuator, the dynamic test was performed, but the change in relative resistance was now −0.75, which is slightly higher than that used for the tendon-based actuator (Figure 10a).

After the partial cut of the elastomeric matrix (Figure 10d), the change in relative resistance was decreased to 0.6. The use of piezoresistive sensors based on carbon fillers under compression mode has not been investigated in the literature. Therefore, further investigations have to be performed in order to better understand the sensor behavior under compression mode. After healing (Figure 10e), the value of the relative resistance partially recovered, and the drift that was seen after the damage disappeared (Figure 10c). Similar to the tendon-based actuator, the *R*_0_ increased after the damage, from 498 kΩ to 605 kΩ. After the healing, the value dropped to 474 kΩ.

**Figure 11:**
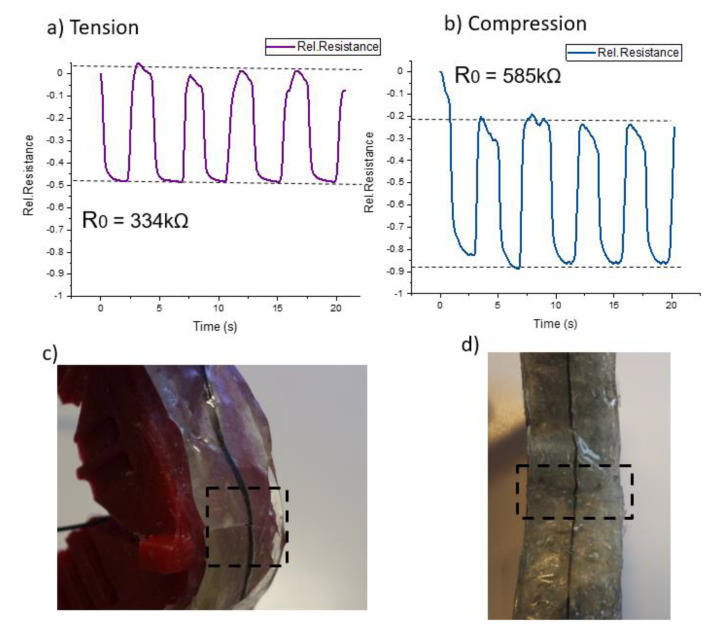
The electrical signal of the piezoresistive skin attached on (**a**) the tendon-based actuator and (**b**) the pneumatic-based actuator after healing the two cut parts of the composite in (**c**) the tendon-based actuator and (**d**) the pneumatic-based actuator.

In addition, the electronic ShSFC skin was cut to completely damage the sensor fiber. Due to the complete severing of the sensor fiber, it was no longer possible to record the electrical sensor signal. After healing, the sensor signal could be restored (Figure 11a and b) by heating the ShSFC to 95 °C for 10 min to achieve self-healing in the matrix and the sensor fiber. Because of the melting temperature (180 °C) of the thermoplastic elastomer-based actuator structure, the structural integrity of the robotic bending modules was not endangered. Similarly, the sensorized skin was able to regain its mechanical and electrical properties while maintaining structural integrity. Because of the adhesive used, it was possible to retain the pre-straining conditions during damage and after healing.

In the tendon-based actuator, the relative resistance (0.48) returned to the initial value (0.50), and only a scar was visible on the surface of the matrix (Figure 11c). After the healing, no significant drift of the sensor signal could be observed. For the pneumatic-based actuator, the relative resistance change (0.69) returned to its initial value (0.70). Again, a small scar could be seen after the healing (Figure 11d), and a small drift after the fifth cycle of the sensor signal could be observed. In this case, the *R*_0_ did not recover after the healing in the two actuators. In the tendon-based actuator, the value was lower after the healing (334 kΩ), and in the pneumatic-based actuator it was higher (585 kΩ). One reason for this change in the resistance value could be that heating the composite above the softening temperature caused a rearrangement of the conductive network inside the composite. This rearrangement had the opposite effect on the *R*_0_ for the two actuators. Overall, the sensor response of the piezoresistive skin in the tendon-based actuator showed less drift and relaxation after the self-healing process.

**Figure 12:**
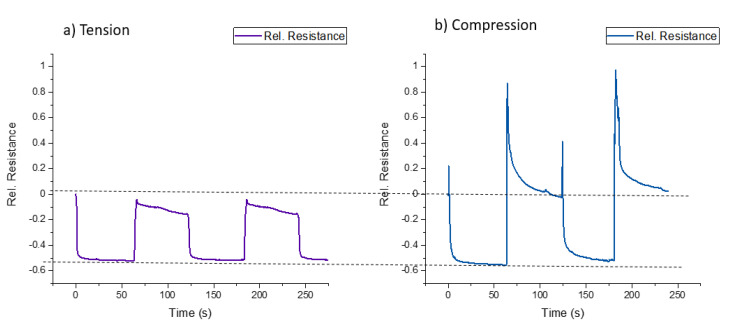
The electrical signal of the piezoresistive skin attached to (**a**) the pneumatic-based actuator and (**b**) the tendon-based actuator during the quasi-static testing, with a dwell time of 60 s at the points of maximum and minimum deformation.

In addition to the cyclic test, quasi-static testing, with a dwell time of 60 s at maximum and minimum deformation for the soft robotic bending actuator, was carried out on the ShSFC-sensorized skin (Figure 12). The purpose of this test was to assess the relaxation of the electrical signal, as described by Georgopoulou et al. [31].

In the case of the pneumatic-based bending actuator (Figure 12a), an overshoot was observed when the actuator changed its position. This overshoot resulted in a significant change in the sensor signal, of 190%. In the case of the tendon-based actuator (Figure 12b), no overshoot was present. Values of 40% and 8% relaxation were calculated at the positions of minimum and maximum deformation, respectively. For the sensor composite of this study, the design considerations require a sensor response with significantly lower relaxation and better damage detection.

## 4. Conclusions

In this study, a self-healing sensor fiber (ShSF) was made via the combination of a supramolecular self-healing elastomer and carbon black. The ShSF showed a monotonic piezoresistive response, when a pre-strain of 50% was applied. The pre-straining also helped to eliminate the buckling that appeared at strains below 50%. Due to the small diameter of the ShSF (0.5 mm), it was not practically feasible to evaluate the self-healing properties of the fractured ShSF alone, given the difficult alignment of the ShSF. For that reason, the fiber was embedded in a self-healing elastomer matrix, using the lamination method, and the resulting self-healing sensor fiber composite (ShSFC) showed no noise and linear response even at low strains. Nonetheless, in that case, there was significant drift in the sensor signal. Pre-straining at 20% (above the buckling area) helped to reduce the drift, but the buckling did not disappear, which was an effect of the viscoelasticity of the elastomeric matrix of the ShSFCs.

The ShSFCs were used in the application of sensorized electronic skin to soft robotic bending actuator modules. Two different robotic modules were examined—a tendon-based actuator that exerted tension on the attached electronic skin, and a pneumatic-based actuator that exerted compression on the electronic skin. Damage inflicted on the elastomeric matrix could be detected in both robotic modules due to the appearance of significant drift in the sensor signal, resulting from the fracture in the matrix caused by the cutting. After the self-healing of the elastomeric matrix at room temperature, the change in relative resistance was recovered for the tendon-based actuator. Moreover, the drift disappeared in both cases, signaling successful healing. When catastrophic damage was inflicted on the self-healing fiber itself, heating to the melting point was necessary to ensure healing. This effect showed that including carbon black in the ShSF influenced the self-healing properties of the systems, and future work could look into conductive fillers that would allow the healing process at room temperature. Similar to the damage of the elastomeric matrix only, the drift could be used to detect this kind of damage, and this drift disappeared after the healing. The value of the resistance changed somewhat after the healing of the ShSF, and therefore a new calibration was required after healing.

Overall, our piezoresistive electronic skin allowed the detection of damage and the monitoring of the healing of fractures and tears. Although the piezoresistive skin showed a linear response in terms of both compression and tension deformation, in compression, greater relaxation was observed together with the partial recovery of the relative resistance after the healing. If these design considerations are taken into account, then the self-healing piezoresistive skin based on ShSFC can find further application in increasing the robustness of soft robotic systems, thereby improving their efficiencies.

## Figures and Tables

**Table 1:** The mechanical properties of the self-healing supramolecular elastomers UPy-1, UPy-2 and UPy-1 with carbon black filler, before damage and after 4 h of healing at room temperature (RT). The data are derived from Supplementary Figures S1 and S2.

Elastomer	Young’s Modulus (MPa)		Ultimate Tensile Strength (MPa)		Strain at the Point of Fracture (%)	
	Before Damage	4 h Healing (RT)	Before Damage	4 h Healing (RT)	Before Damage	4 h Healing (RT)
UPy-1	0.6	0.7	1.2	0.9	1200	710
UPy-1 30% CB	9.5	13	2.2	1.6	70	26
UPy-2	3.3	3.3	8.3	8.3	900	900

**Table 2:** Characteristics of the ShSF performance derived from the dynamic tensile testing of the ShSF.

Range of Strains (%)	0–50	50–100
Drift (%)	110	2
Strain at which the slope changes from positive to negative (%)	46	No change
Strain at which buckling occurs (%)	6	No buckling

**Table 3:** Characteristic values of the electrical sensor performance derived from the dynamic tensile testing of the ShSFC. The initial resistance was calculated as the mean value from five samples.

Range of Strains (%)	0–50	25–75
Drift (%)	63	22
Buckling at strains (%)	24	49
Initial Resistance (kΩ)	511 ± 4	

## Data Availability

Not applicable.

## Supplementary Materials

The following are available online at https://www.mdpi.com/article/10.3390/polym13172983/s1, Figure S1: Tensile curves for the UPy elastomer 1, Upy elastomer 2 and the composite of UPy elastomer 1 with 30 wt.% carbon black. Figure S2: Stress-elongation curve up to the point of fracture for the elastomer UPy-1 and the composite UPy-1 with 30% carbon black before damage and 4 h after healing.
