# Supplementary material for: Supramolecular Self-Healing Sensor Fiber Composites for Damage Detection in Piezoresistive Electronic Skin for Soft Robots
Source: Polymers (Basel). 2021 Sep 2;13(17):2983. doi: 10.3390/polym13172983 (PMC8433753; doi:10.3390/polym13172983)
Supplement: Supplementary file 1 [file polymers-13-02983-s001.zip › polymers-1347501-supplementary.pdf]

Supplementary Figures

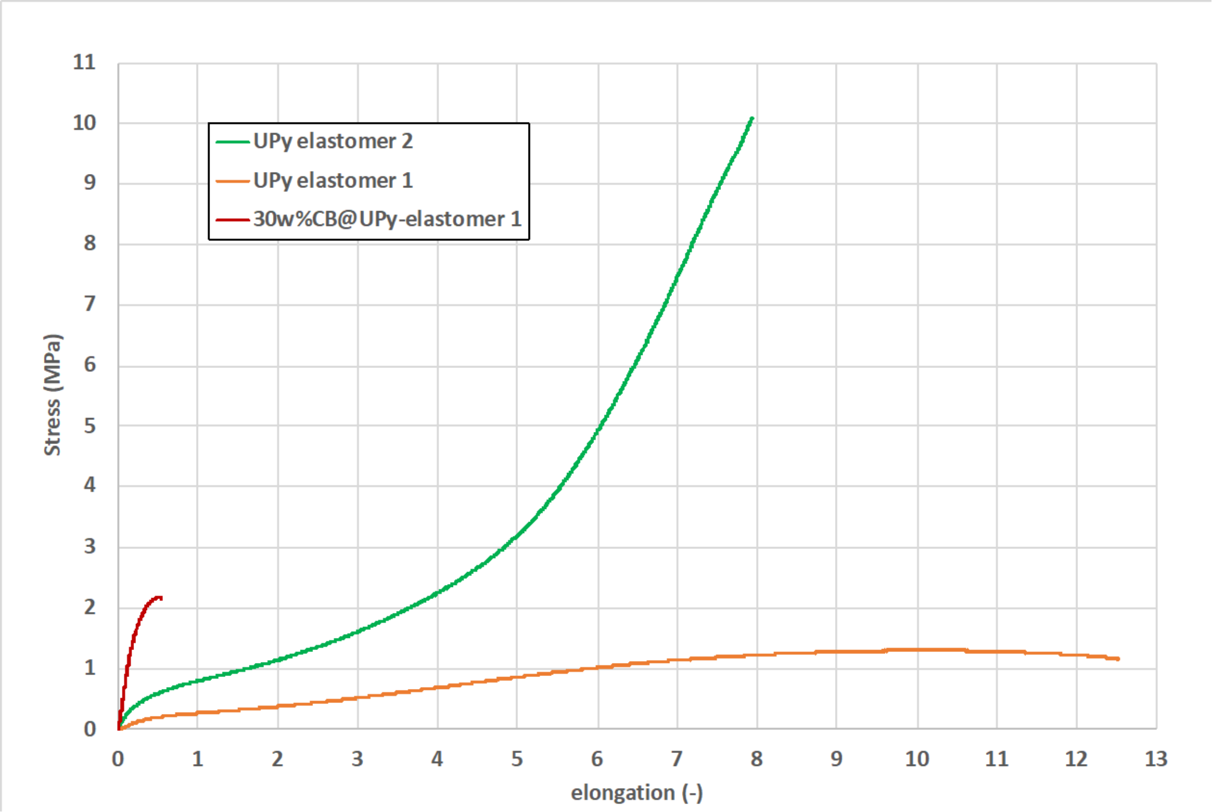

Figure S1. Tensile curves for the UPy elastomer 1, Upy elastomer 2 and the composite of UPy elastomer 1 with 30 wt.% carbon black.

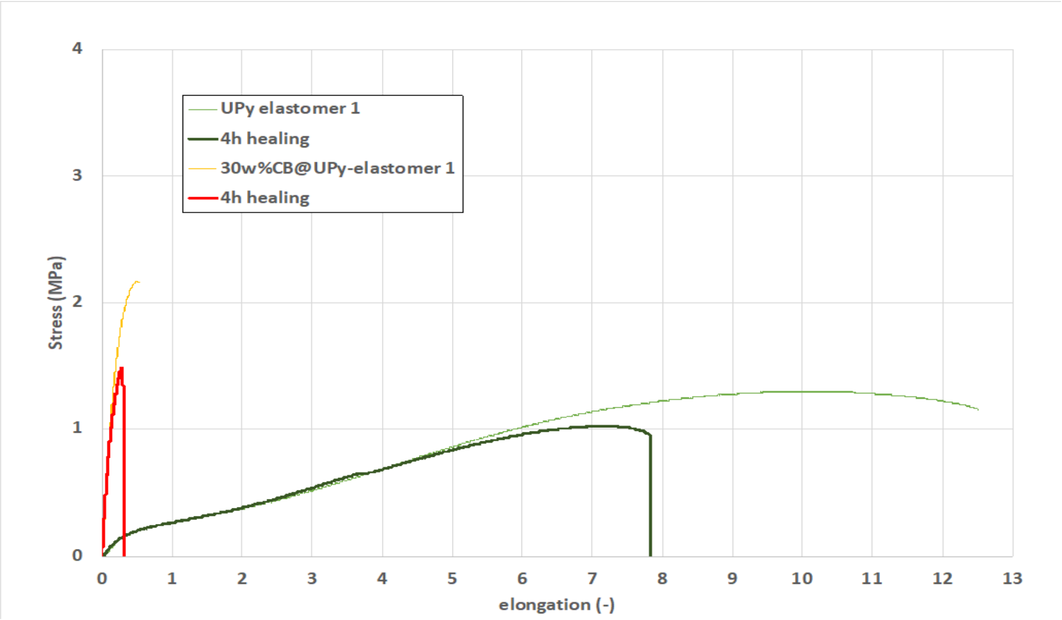

Figure S2. Stress-elongation curve up to the point of fracture for the elastomer UPy-1and the composite UPy-1 with 30% carbon black before damage and 4 hours after healing.
